# Exogenous Schwann Cells Migrate, Remyelinate and Promote Clinical Recovery in Experimental Auto-Immune Encephalomyelitis

**DOI:** 10.1371/journal.pone.0042667

**Published:** 2012-09-11

**Authors:** Violetta Zujovic, Cédric Doucerain, Antoine Hidalgo, Corinne Bachelin, François Lachapelle, Robert Weissert, Christine Stadelmann, Chris Linington, Anne Baron-Van Evercooren

**Affiliations:** 1 Université Pierre et Marie Curie-Paris 6, Centre de Recherche de l′Institut du Cerveau et de la Moelle Epinière, UMR-S975, Paris, France; 2 Inserm, U 975, Paris, France; 3 CNRS, UMR 7225, Paris, France; 4 AP-HP, Hôpital Pitié-Salpétrière, Fédération de Neurologie, Paris, France; 5 Department of Neurology, University of Regensburg, Regensburg, Germany; 6 Institut für Neuropathologie, Göttingen, Germany; 7 Institute of Infection, Immunity and Inflammation, University of Glasgow, Glasgow, United Kingdom; Washington University, United States of America

## Abstract

Schwann cell (SC) transplantation is currently being discussed as a strategy that may promote functional recovery in patients with multiple sclerosis (MS) and other inflammatory demyelinating diseases of the central nervous system (CNS). However this assumes they will not only survive but also remyelinate demyelinated axons in the chronically inflamed CNS. To address this question we investigated the fate of transplanted SCs in myelin oligodendrocyte glycoprotein (MOG)-induced experimental autoimmune encephalomyelitis (EAE) in the Dark Agouti rat; an animal model that reproduces the complex inflammatory demyelinating immunopathology of MS. We now report that SCs expressing green fluorescent protein (GFP-SCs) allografted after disease onset not only survive but also migrate to remyelinate lesions in the inflamed CNS. GFP-SCs were detected more frequently in the parenchyma after direct injection into the spinal cord, than via intra-thecal delivery into the cerebrospinal fluid. In both cases the transplanted cells intermingled with astrocytes in demyelinated lesions, aligned with axons and by twenty one days post transplantation had formed Pzero protein immunoreactive internodes. Strikingly, GFP-SCs transplantation was associated with marked decrease in clinical disease severity in terms of mortality; all GFP-SCs transplanted animals survived whilst 80% of controls died within 40 days of disease.

## Introduction

The development of clinical deficits in multiple sclerosis (MS) is due to repeated episodes of inflammatory demyelination that result in axonal loss and formation of persistently demyelinated plaques of gliotic scar tissue. Enhancing remyelination by transplanting myelin forming cells into the CNS is predicted to provide significant clinical benefits, but as yet there is no consensus as to which cells to use, or the best route of delivery [1].

However, Schwann cells (SCs), the myelinating glia of the peripheral nervous system (PNS) exhibit a range of properties that make them attractive candidates for cell therapy in MS[1]. Not only can large numbers of SCs be obtained by *in vitro* expansion [2]–[5], but in a variety of experimental settings they not only remyelinate demyelinated lesions [6], but restore conduction [7] and promote functional recovery [8]. Autologous transplantation of SCs in primates has been proven feasible and successful in terms of CNS remyelination [9]. Moreover, spontaneous SC remyelination does occur in some cases of MS [10]–[13], an observation demonstrating that despite ongoing disease activity the CNS can support SC mediated repair.

Recent data indicate that exogenous PNS stem/precursor cells are of greater interest for myelin repair than committed SCs due to their greater ability to interface with astrocytes and migrate within the CNS parenchyma [14], [15]. More recently, reports that SCs can be derived from multipotent or pluripotent stem cells in embryonic and adult tissue [14]–[19] or via iPS technology [20]– opened new perspectives for developing SC-based treatments for demyelinating disorders of the CNS. However, this strategy must be reassessed after a first phase I clinical trial failed to provide evidence for the survival of autologous SC transplanted into MS lesions (http://www.myelin.org/0623003.htm).

We therefore investigated the behaviour and survival of SCs transplanted into the CNS of Dark Agouti (DA) rats with myelin oligodendrocyte glycoprotein (MOG)-induced experimental allergic encephalitis (EAE). This animal model of MS reproduces the histopathology and clinical course of the human disease. In particular animals develop inflammatory demyelinating lesions in the spinal cord that ultimately form confluent plaques of chronically demyelinated gliotic scar tissue, the pathological hallmark of MS [24]–[26]. We report that transplanted SC not only survive and differentiate into myelinating glia in this pathological environment, but that this is associated with reduced clinical disease severity relative to sham operated controls.

## Results

### Characterization of transduced SCs

**Figure 1:**
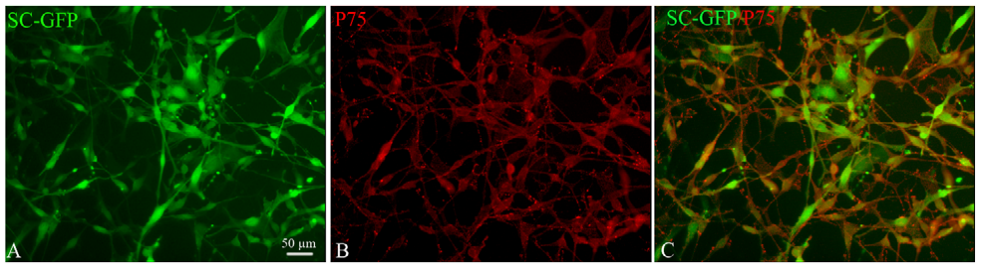
*In vitro* characterization of transduced SC-GFP. The majority of SC-GFP (green, A, C) express the SC marker p75 (red, B, C).

In order to trace transplanted SC, we transduced DA rat SCs with a lentiviral vector to induce cytoplasmic expression of GFP (Fig 1A, 90% of GFP expressing SC). Immunocytochemistry for the SC specific marker p75 confirmed that GFP expression did not modify SCs phenotype, more than 95+/2 % of the cells in both GFP-SCs and non-transduced SCs expressed p75 (Fig 1B, C).

### Transplanted GFP-SCs survive and migrate in the CNS parenchyma

**Figure 2:**
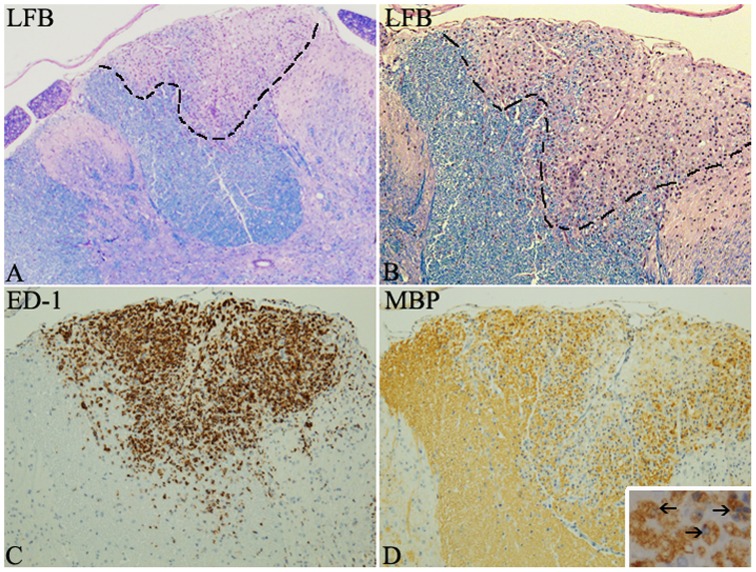
Illustrations of a focal spinal cord lesion of MOG induced EAE in the DA rat. LFB staining shows a focal demyelinated lesion in the dorsal funiculus with myelin loss (A–B). ED-1 immunostaining highlights the presence of macrophages/microglia cells within the lesion (C). MBP immunohistochemistry illustrates the loss of myelin and the presence of myelin-laden macrophages (close up in insert) is indicative of recent myelin phagocytosis (D).

**Figure 3:**
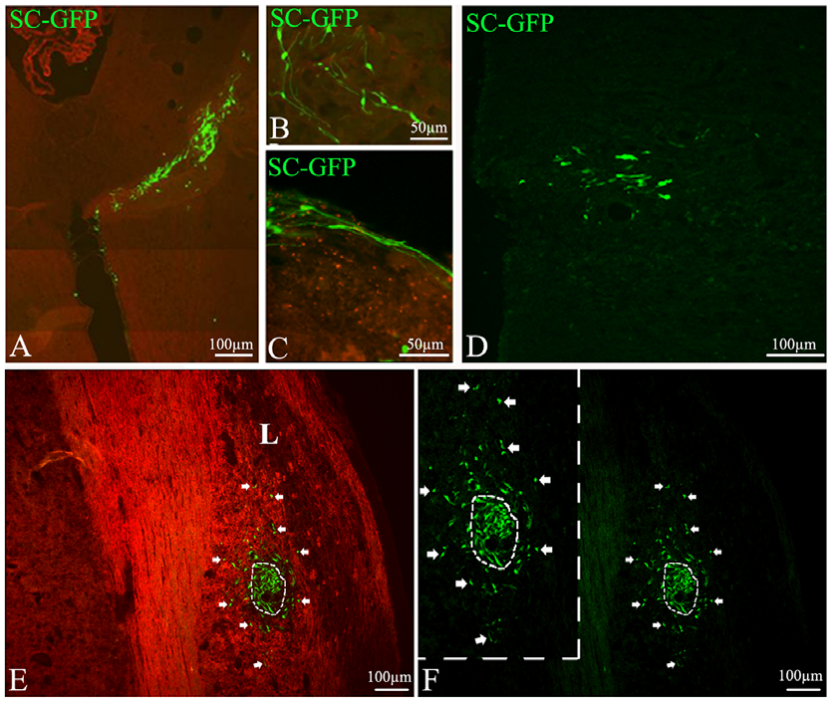
Distribution of GFP labeled SC after delivery in the cisterna magna and the spinal cord. GFP-SC (green) are detected both in the cerebellar parenchyma (A, B) and meninges (C) 7 days (A–C) after cisterna magna delivery as well as 21 days after in the proximal spinal cord (D). GFP-SCs grafted in the spinal cord parenchyma (E, F) are concentrated around blood vessels, some migrate away from the graft toward a lesion (L) identified by MOG immunostaining through white matter (E, arrows). (F) Same field illustrating GFP-SCs, inset is a higher magnification.

The effect of inflammatory demyelination on SC migration and differentiation was studied by allografting GFP-SCs into the CNS of DA rats with MOG-induced chronic EAE [24]. In this model, inflammatory demyelinated lesions characterized by luxol fast blue (LFB) and anti-MBP to detect demyelination, and ED-1 to detect inflammatory cells are heterogeneously widespread throughout the CNS including the spinal cord as previously described [24] and as illustrated in Fig. 2. Since initial signs of disease (loss of weight and tail tone) were typically observed 11–12 d.p.i., SCs were grafted 2 to 4 ays later. Two delivery routes were investigated: (1) injection into the cerebrospinal fluid via the cisterna magna; (2) direct injection into the spinal cord parenchyma. Animals were sacrificed at 7 and 21 days post grafting, and brain and spinal cord screened to evaluate survival and location of the grafted cells. GFP-SCs were detected in the CNS parenchyma after both delivery modes and at both time points (Fig 3 and Fig S1). GFP-SCs injected into the cerebrospinal fluid at the level of the cisterna magna were often localized in the cerebellum (75%) (Fig 3 A–C) but were also detected in the cervical spinal cord (25%) (Fig 3D) indicating their ability to migrate over 2 mm from the injection point. In contrast GFP-SCs grafted directly into the spinal cord remained concentrated in the vicinity of the injection site after 7 days (Fig 3E, Fig S1A) but migrated over quite some distance (1.2 mm) after 21 days (Fig S1B).

**Figure 4:**
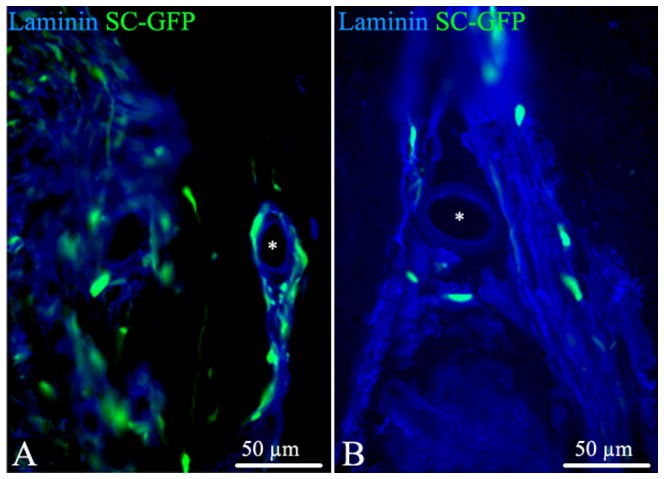
One route of SC migration: the blood vessels. SC grafted in the spinal cord parenchyma are often localized in white matter around blood vessels (asterisks), evidenced with anti-laminin antibody (blue). While at 7 days SC are present close to the blood vessel wall (A), at 21 days they are embedded in the perivascular space but remote from the vascular wall (B).

Irrespective of the route of delivery GFP-SCs populated areas of both grey (Fig 3A, B) and white matter (Fig 3E). Seven days post-delivery, the cells were oriented randomly but within 21 days tended to become aligned suggesting they may be interacting with axons and differentiating into myelin-producing cells (Fig. S1). After direct injection into the spinal cord parenchyma large numbers of GFP-SCs were also observed in close apposition with blood vessel wall (Fig. 4 A, B). However whilst still present in the perivascular space 21 days later they had by now migrated away from the blood vessel into the perivascular matrix (Fig 4B); an observation suggesting this basement membrane provides a route by which SCs can migrate through the CNS.

### SCs are recruited by demyelinated lesions and mix with astrocytes

**Figure 5:**
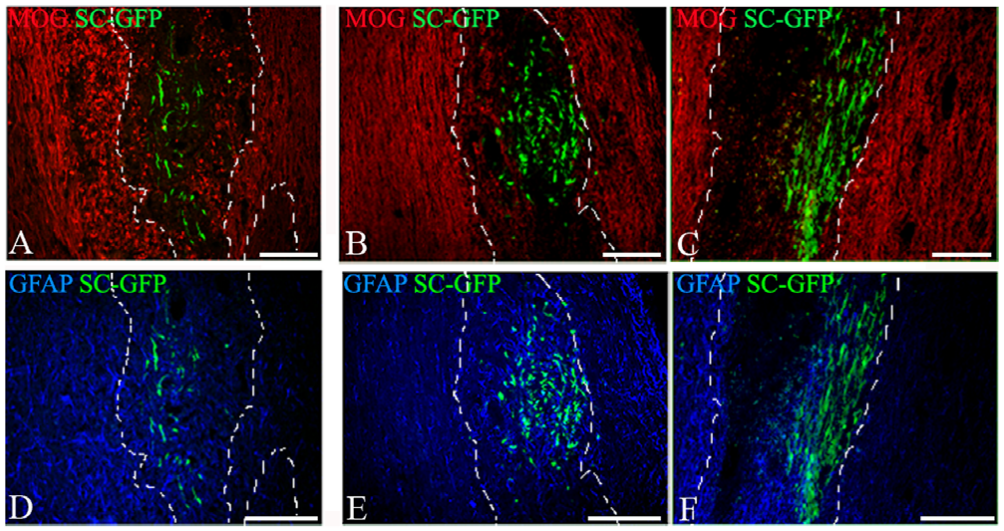
Interaction of GFP-SCs with myelin and astrocytes after delivery in the cisterna magna or the spinal cord parenchyma. Combined detection of MOG, GFAP and GFP on cryostat sections showed that GFP-SCs grafted in the cisterna magna (A, D) or the spinal cord (B,C, E, F) are found in demyelinated lesions (delineated by dashed lines) detected by MOG immunostaining (red) both after 7 days (B) and 21 days (A, C) after delivery. GFAP+ astrocytes (blue) interact with GFP-labeled SCs in the cisterna magna (D) and the spinal cord (E, F); 7 days (E) or 21 days (F) after spinal cord graft or 21 days after cisterna magna graft (D).

In DA rats with MOG-EAE, inflammatory demyelinating lesions develop throughout the CNS including cerebellum and spinal cord [24], a feature that allowed us to investigate the ability of transplanted SC to populate and/or myelinate lesions at sites remote from the initial site of delivery. Immunostaining for MOG revealed that GFP-SCs were recruited selectively into demyelinated lesions compared to the surrounding parenchyma irrespective of the route of delivery (Fig 5A–C). This observation highlights that SCs not only survive in this inflammatory demyelinating environment, but also selectively populate areas of demyelination.

The ability of transplanted SCs to populate these lesions was somewhat surprising as inflammatory demyelination in EAE is associated with astrocytosis and the formation of glial scar tissue. In other experimental settings this astrocytic response is reported to inhibit SCs migration [8], [27], [28]. Immunolabeling for the astrocytic marker GFAP revealed that this was not the case in animals with MOG-EAE. In this case transplanted GFP-SCs were observed to be intimately intermingled with GFAP^+^ astrocytes in demyelinated lesions both at 7 (Fig 5D, E) and 21 days post transplantation (Fig 5F).

### Grafted SCs participate more effectively to remyelination after intra-parenchymal spinal cord delivery than intra-thecal delivery

**Figure 6:**
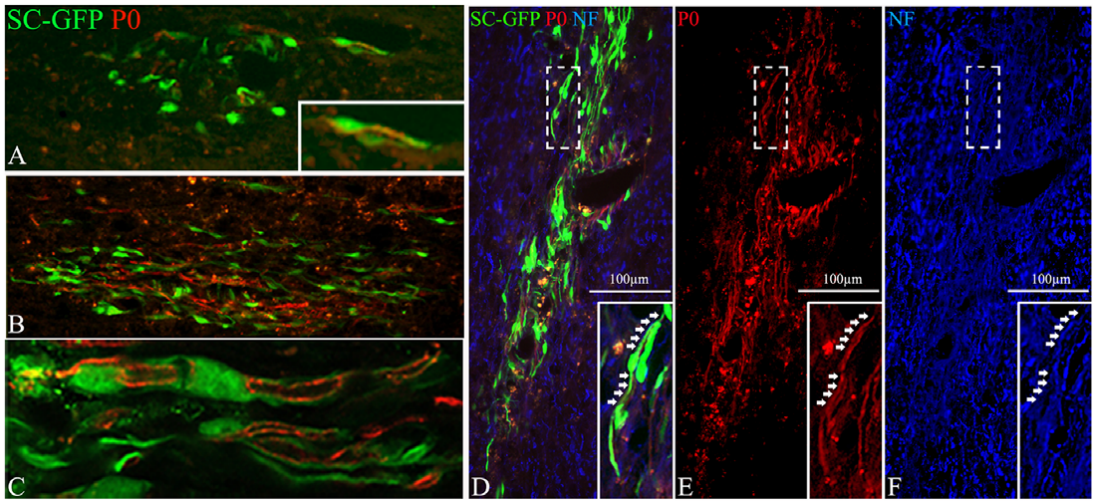
Comparison of the remyelination potential of GFP-SCs grafted in the cisterna magna or the spinal cord. Twenty one days after SC delivery in the cisterma magna (A) or the spinal cord (B–F), exogenous remyelination was assessed using an anti-P0 antibody (red). P0 positivity colocalized with GFP-SCs (C, confocal microscopy), more frequently after spinal cord graft than cisterna magna delivery. C is a higher magnification of B and corresponds to the lesion detected on an adjacent section and illustrated in Fig 5C. GFP-SCs produce P0+ myelin (red D, E) that surrounds neurofilament (NF) + axons (blue D, F). Inserts are higher magnifications of dashed boxes.

The above studies demonstrated that in contrast to chemically induced demyelinated spinal cord [28], mature SCs readily migrate to populate inflammatory demyelinating lesions in animals with EAE. To determine if these SCs are then able to participate in remyelination, we selected sections adjacent to those where GFP+ cells were identified in MOG deprived lesions, and performed immunolabelling for P0, a specific marker of peripheral myelin and investigated its co-localization with GFP and neurofilament (Fig 6). Although characteristic P0 + railroad like myelin tracks (Fig 6C) were observed in all animals in which GFP-SCs were engrafted directly into the spinal cord parenchyma (Fig 6B–F), these were only observed in 25% of the animals in which GFP-SCs were introduced via the cisterna magna (Fig 6A). The contribution made by the grafted GFP-SCs to P0 remyelination also depended on the route of delivery. In animals receiving grafts directly into the spinal cord >25% of P0 immunoreactivity was associated with GFP expression (Fig 6 B–F). However in animals receiving GFP-SCs via the cisterna magna less than 10% of P0 immunoreactivity was associated with GFP expression (Fig 6A). These observations demonstrate that not only does SCs mediated repair occur in this model of MS, it is significantly enhanced by intra-parenchymal SCs transplantation.

### SC graft effect on EAE score evolution

**Figure 7:**
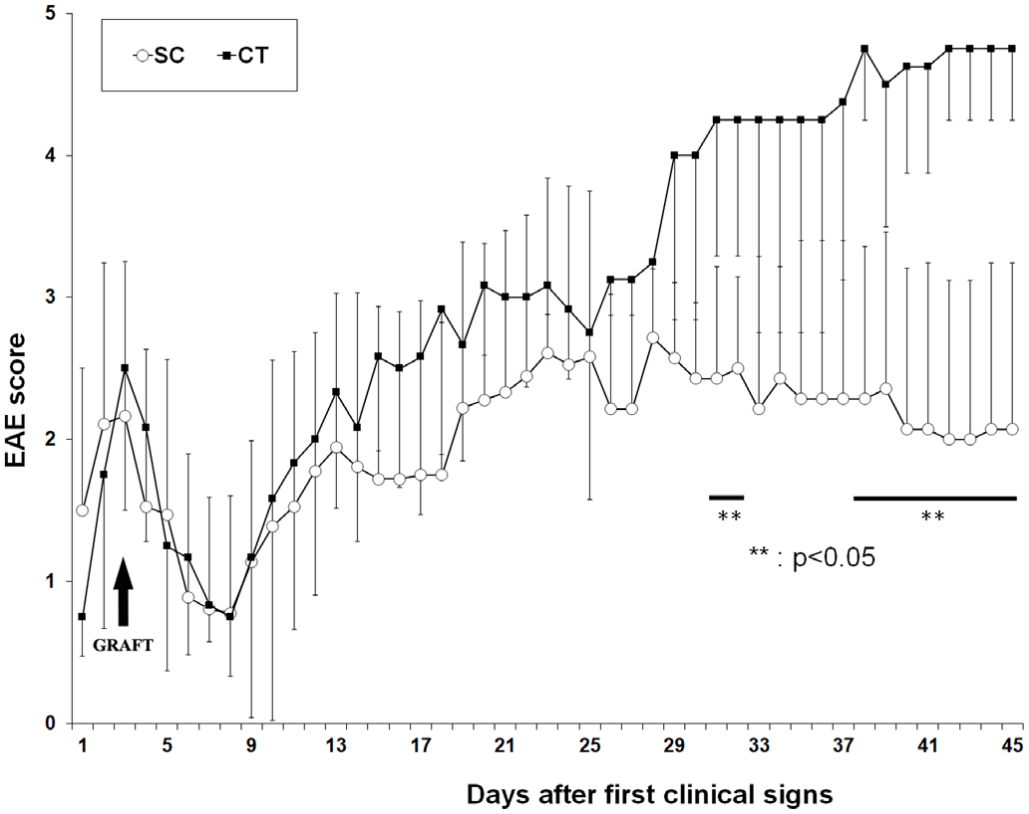
EAE score evolution after GFP-SC graft in the spinal cord. The graph depicts the clinical scores of EAE animals grafted with GFP-SC (white circles, 7 animals) and medium injected animal (black squares, 5 animals). Surgeries were performed 2 days after the first clinical signs (occurring 12 days after the induction of the disease). A difference between the two groups is observed from day 25 and is significant (Mann-Whitney rank test; p<0.05) around 30 days after the first clinical signs.

Since remyelination by GFP-SCs was found most efficient following direct injection into the spinal cord parenchyma, this was repeated to investigate if this protocol would influence the clinical course of disease. Disease activity in animals receiving GFP-SCs (n = 7) was compared to that in a group of sham operated controls (n = 5) animals and non-operated animals (n = 9) for 45 days following the first clinical signs of disease. No significant differences were observed in the first 25 days after disease onset, but from day 31 on, the SCs injected group began to show significant improvements relative to the controls (Fig 7). Furthermore, SC-injected-animals less severe disease progression was confirmed with respect to the cumulative disease score and mortality compared to sham operated and non-operated animals (Table 1). In contrast to sham operated controls and non-operated animals, whose survival rate was in average 25%, disease progression in animals receiving GFP-SCs transplants was halted from day 25 onwards and none died.

**Table 1:** Average scores, maximal scores, cumulative scores (calculated from day 25 to day 45) and mortality rate in non-operated, CT and SCs grafted animals.

	NON-OPERATED (9 animals)	CONTROL (5 animals)	SC (7 animals)
Average score	3.36±1.12	2.72±0.68	1.86±0.63
Score max	4.37±0.91	4.33±0.81	3.31±0.44
Cumulative scores (25–45)	88.4±23.4	88.1±16.2	53.5±8.71 *
Survival	33%	20%	100 *

* : is significantly different from control and non-operated animals, p<0.05.

## Discussion

A recent phase I clinical trial of autologous SCs transplantation in MS was discontinued after brain biopsies performed five months after transplantation failed to provide evidence for SCs survival. This is in stark contrast to the situation in experimental models of toxin-mediated demyelination in which the transplanted SCs not only survive but actively participate in lesion repair in the CNS including in conditions of autologous transplantation in large primates. To better understand the behavior of SCs in the inflammatory demyelinating environment of the MS lesion we chose to follow the fate of allogenic SCs transplanted into DA rats with MOG-induced EAE, a chronic relapsing disease model that reproduces many aspects of the complex clinical course and pathology of MS [24]. Our data demonstrate exogenous SCs not only survive in the chronically inflamed CNS, but populate demyelinating lesions where they can differentiate and myelinate CNS axons. This is associated with a significant decrease in disease severity, but at this time it is not possible to state whether this is due to SCs mediated repair *per se*, or an direct or indirect immunomodulatory effect. These results demonstrate that SCs transplantation has a beneficial clinical effect in this model of MS, but also that inflammatory demyelination generates an environment favorable to SCs survival, migration and differentiation.

Several elements indicate that exogenous SCs behaved differently after transplantation in EAE compared to non-inflammatory models of demyelination. We show that in diffuse EAE, SCs are found along meninges, and associated with perivascular spaces (Fig. 4). In addition and unlike in focal toxin models of demyelination or trauma, exogenous SCs seemed to progress through white matter and grey matter, substrates which are highly inhibitory to SCs migration *in vitro* and *in vivo* [28], [29].While it was difficult to appreciate SCs migration when SCs were directly grafted within the spinal parenchyma of EAE animals given the wide-spread dissemination of lesions, the presence of SCs in the cervical spinal cord after intra-thecal delivery, clearly indicated SCs migration from the point of injection (cisterna magna) through the parenchyma to reach the cervical spinal cord. Moreover, while in toxin-induced models of demyelination, exogenous SCs are restricted to astrocyte free areas [30], we found that in EAE lesions, SCs mixed entirely with astrocytes without the formation of a noticeable scar.

We also demonstrated that recruited exogenous SCs differentiated in myelin competent cells as confirmed by their progressive polarization along CNS axons and the detection of Pzero-positive myelin-like internodes. Robust remyelination of central axons by the grafted SCs occurred in the majority of animals, indicating that inflammation does not appear to prevent SCs differentiation. However, myelin repair by exogenous SCs was more frequent after intra-parenchymal than intra-thecal delivery. This could be explained by the fact that transgression of the pia mater may delay their intra-CNS migration and recruitment by the lesion, and consequently decrease their involvement in myelin repair. Thus such as in toxin-induced model of demyelination, the myelination efficiency of exogenous SCs is highly depending upon the rapidity by which the grafted cells arrive at the lesion-site and compete with endogenous cells [28]. In focal toxin lesions, remyelination of CNS axons by SCs, is generally confined to the center of the lesion where astrocytes are absent. By contrast, in EAE, remyelination by exogenous SCs appeared to be spread over the lesion, and among GFAP+ astrocytes correlating with the better mixing of astrocytes and exogenous SCs. These data indicate that in chronic EAE, inflammation has a beneficial effect on exogenous SCs integration/migration in the CNS and does not impede remyelination of CNS by exogenous SCs.

How inflammatory demyelination supports the survival, recruitment and differentiation of exogenous SCs in this EAE model remains an open question. Immune mediated loss of MOG^+^ oligodendrocytes within the developing lesion clearly provides SCs an opportunity to myelinate axons in the absence of competition from endogenous oligodendrocytes. Whilst remodeling of the lesion environment may reduce expression of factors known to inhibit SCs migration such as Semaphorin 3A (Sema 3A) [31], N-Cadherin [32], CSPG [33] and Ephrin-EphR interactions [34], many other molecular cues known to influence SCs proliferation and/or differentiation are also up regulated in the inflamed CNS [35], [36]. These include TGF-beta 1, a positive regulator of SCs polarization [37] and myelination [38] and secretory leukocyte protease inhibitor (SLP1) which promotes differentiation of neural stem cells and oligodendrocytes by repressing the myelination inhibitor HES1 [36], [39]. Crucially, not only is HES1 also a negative regulator of SC differentiation [40], SLP1 is over expressed in MOG-EAE [36] raising the possibility that this might support the differentiation of exogenous SCs into myelin-forming cells within demyelinating lesions. These effects may explain why SCs can populate and differentiate into myelinating cells within inflammatory demyelinating lesions, but do not account for the recruitment of SCs from the transplantation site. This may be explained by SCs expression of CXCR4 and CXCR7 which are receptors for CXCL11 and CXCL12; two chemokines highly up regulated in EAE [41] and MS [42]. Currently little is known about the functional significance of these receptors regards SCs chemotaxis, but CXCL12 signaling induces SCs proliferation [43], and abolishes the inhibitory effects of Sema 3A [44].

In the current study we noticed that clinical amelioration of disease activity was only observed approximately 31 days after disease onset. It was not possible to correlate this effect with the extent of immunoreactivity for P0 protein in these animals suggesting that the reduction in clinical deficits compared to controls is not simply due to remyelination. This indicates that clinical recovery is mediated by other mechanisms. SCs are known to secrete a variety of neurotrophins including nerve growth factor (NGF), neurotrophine-3 (NT3), brain-derived neurotrophic factor (BDNF), fibroblast growth factor (FGF), glial cell line-derived neurotrophic factor (GDNF) and ciliary neurotrophic factor (CNTF) [45], responsible of their role in axonal survival and regeneration. Moreover, over-expression of NT3 and BDNF by exogenous SCs had multiple effects in a toxin model of spinal cord demyelination including promoting endogenous CNS remyelination, reduction of neurodegeneration and glial scarring in correlation with improved functional recovery [8]. It is therefore possible that exogenous SCs exert a trophic effect on endogenous glial or neuronal cells as previously demonstrated after delivery of neural precursor cells in EAE mice [35], [46]. Clinical recovery can also be induced via immunomodulatory properties of the gratfed cells such as NPCs [46], [47]. SCs can modulate immune response via expression of Fas ligand (FasL) at their surface, a ligand whose interaction with its receptor Fas induces lymphocyte apoptosis. Consequently, SCs were found to contribute to the diminution of immune attacks in a model of experimental auto-immune neuritis by participating to the elimination of invading auto-reactive T cells in the PNS through their expression of Fas L [48]. Interestingly, FasL can also act as a reverse signal-transducing molecule in SCs, leading to the secretion of NGF, and enhanced peripheral nerve regeneration [49]. Although not investigated, such mechanisms may have operated after SCs engraftment in EAE.

SCs based therapy is a prominent field of investigation for trauma and demyelinating diseases such as MS. Our findings show that SCs behavior in inflammatory conditions sheds the light on novel SCs function, providing anatomical and clinical recovery in a rodent model of MS without being the target of recurrent immune-mediated demyelination, which characterizes MS pathology.

## Materials and Methods

### Induction and clinical scoring

Eight week-old DA female rats (Janvier) were housed in the Pitié-Salpêtrière EOPS animal facility. All animal protocols were performed in accordance with the guidelines published in the National Institute of Health Guide for the Care and Use of Laboratory Animals. The animal studies described in this work were performed under and approved by the French Agricultural Ministry-Animal Welfare license numbers B75-13-08 and A75–585. Chronic demyelination was achieved by injecting sub-cutaneously 100 µl of a solution of MOG (50 µg) in Complete Freund's adjuvant containing Mycobacterium tuberculosis (H37RA) (225 µg) in the rear footpads and flanks (25 µl/site). Clinical scores were evaluated daily using the following scale: 0 =  healthy, 1 =  tail paralysis, 2 =  ataxia or paresis of hind limbs, 3 =  paralysis of hind limbs and/or paresis of forelimbs, 4 =  tetra-paralysis, 5 =  moribund or death. Mann –Whitney rank test was used to test statistical significance between the different groups.

#### Histology

Rats were transcardially perfused with 4% PFA, brains and spinal cords dissected, and embedded into paraffin. Two micron-thick sections were cut and deparaffinized. To assess inflammation, demyelination luxol fast blue staining (LFB), was performed. In addition, immunohistochemistry was performed with antibodies against CD68 (macrophages, clone ED-1; www.abdserotec.com) and myelin basic protein (MBP; A 0623; Dako, Glostrup, Denmark) as described previously (Herrmann et al., 2005). Diaminobenzidine (www.sigma.de) was used as chromogenic substrate. Sections were counterstained with hematoxylin, dehydrated and mounted with DePeX (www.serva.de). For immunohistochemical stainings, microwave pre-treatment in 10 mM citric acid buffer, pH 6.0, was performed.

### Schwann cells cultures

SCs were purified from adult DA rat sciatic nerves as described previously for adult primate SCs [5]. Purified SCs (95+/−2 %, p75 positive) were expanded and maintained in proliferation medium containing 90% DMEM (Invitrogen, Cergy-Pontoise, France), 10% fetal calf serum (Invitrogen, Cergy-Pontoise, France), 2 µg/ml forskolin (Sigma, St Louis, MO, USA), 10 ng/ml human heregulin ß1 (hHRG-ß1, RetD system) and 10 µg/ml insulin (Sigma, St Louis, MO, USA). To identify SCs in the host parenchyma, adult SCs (5×10^4^) were transduced with 12 ng p24 of HIV-CMV-GFP, which allowed strong and stable cytoplasmic green fluorescent protein (GFP) expression in SCs (GFP-SCs). Expression of GFP was detected 48–72 h post transduction and was stable over time [9], [15].

### SCs transplantation

DA rats were anesthetized with ketamine (100 mg/kg, i.p.) and xylazine (10 mg/kg, i.p.), 2–4 days after the first clinical signs (around 10 days). Allogous GFP-SCs were delivered intra-thecally via the cisterna magna or in the spinal cord parenchyma. For intra-thecal delivery, anesthetized animals were placed in a stereotaxic frame equipped with a Hamilton syringe allowing a direct puncture of the cisterna magna, where 3 µl of SCs suspension (7.10^5^cells/µl) were injected. For intra-parenchymal spinal cord delivery, 2 µl of SCs suspension (5.10^4^ cells/µl) or 2 µl of SCs medium were injected in the dorsal spinal cord white matter funiculus. To evaluate the effect of GFP-SCs injection on the evolution of clinical scores, EAE animals were distributed homogeneously according to their clinical score (average score of 2,3) into 2 groups (n = 5–7 animals per group) before delivery of GFP-SCs or the same volume of medium for controls. Non operated animals induced with EAE were also followed in parallel to the two operated groups.

### Post-mortem evaluation of engrafted EAE animals

Animals were euthanized by a lethal dose of anesthesia (ketamine) and intra-cardially perfused with 2% paraformaldehyde (PFA). The brain and the spinal cord were post-fixed 1 hour in the same fixative, cryo-protected overnight with 20% sucrose and frozen in isopentane cooled by liquid N2. Immunohistochemistry was performed using MOG antibody to detect the area of demyelination, GFAP (rabbit, 1/1000, AbCys, Paris, France) to identify the astrocytes, P0 (Mouse IgG hybridoma, Yoshimura et al., 1996) to identify peripheral myelin and laminin (rabbit, 1/20, Chemicon, Billerica, USA) to localize blood vessels basement membrane. Species-specific secondary fluorescent conjugated antibodies were applied for 1 h in addition to Hoechst 33342 (Sigma,St. Louis, USA). Coverslips were mounted with fluoromount and analyzed under fluorescent DMC Leica microscope (Leica microsystems, Wetzlar, Germany) using Explora Nova Image acquisition software (La Rochelle, France).

## Supporting Information

Figure S1 **Illustration of GFP SCs migration 7 and 21 days after their delivery in the spinal cord.** While at 7 days grafted GFP SCs are detected in the vicinity of the graft (A), 21 days later they spread over a distance of 1.2 mm (B).
(TIF)
