# Supplementary figures and images for: Exogenous Schwann Cells Migrate, Remyelinate and Promote Clinical Recovery in Experimental Auto-Immune Encephalomyelitis
Source: PLoS One. 2012 Sep 11;7(9):e42667. doi: 10.1371/journal.pone.0042667 (PMC3439443; doi:10.1371/journal.pone.0042667)

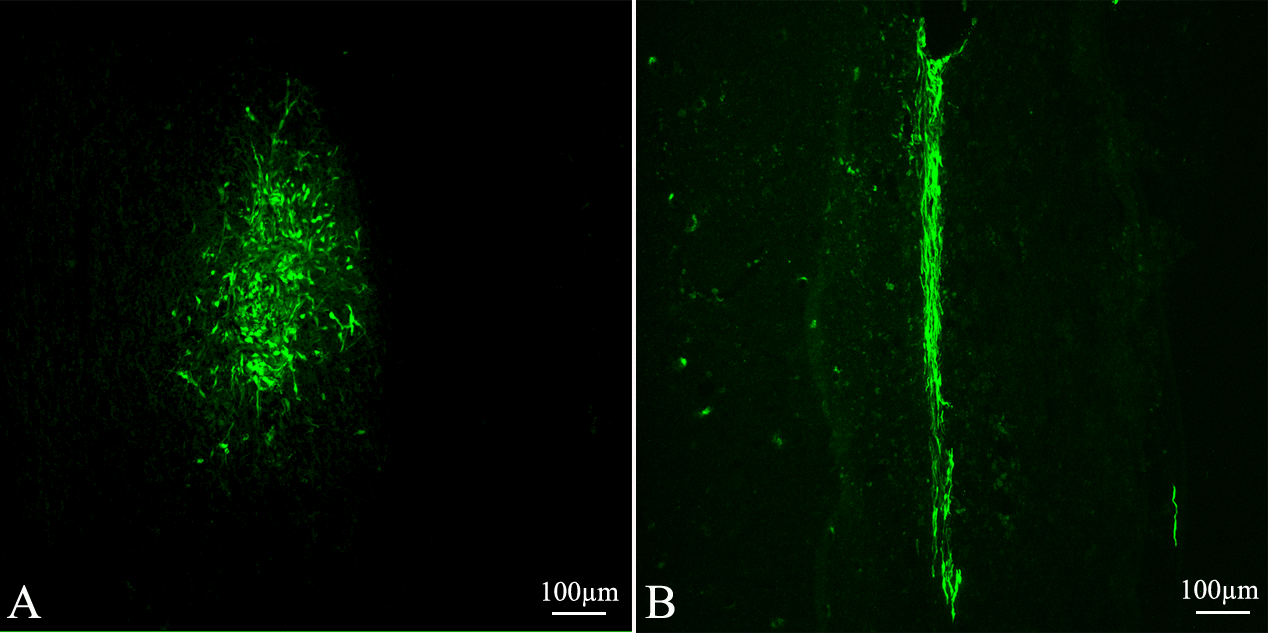

Supplement: Figure S1 — Illustration of GFP SCs migration 7 and 21 days after their delivery in the spinal cord. While at 7 days grafted GFP SCs are detected in the vicinity of the graft (A), 21 days later they spread over a distance of 1.2 mm (B). (TIF) [file pone.0042667.s001.tif]
